# Is rectal preparation necessary in contemporary image-guided prostate radiotherapy?

**DOI:** 10.1016/j.ctro.2026.101207

**Published:** 2026-06-02

**Authors:** S.E. Alexander, L. Booth, L. Delacroix, E. Garrad, A. Gordon, A. Guerra, H. Mohammad, S. Nill, C. Ockwell, U. Oelfke, H.A. McNair, A.C. Tree

**Affiliations:** a The Royal Marsden NHS Foundation Trust, United Kingdom; b The Institute of Cancer Research, United Kingdom; c The Joint Department of Physics, The Royal Marsden Hospital and The Institute of Cancer Research, United Kingdom

**Keywords:** Prostate cancer, Image guided radiotherapy, Patient preparation, Personalised care

## Abstract

**Introduction:**

Rectal preparation during prostate radiotherapy (RT) was established in the era of two-dimensional bone-based IGRT, but its necessity with contemporary three-dimensional IGRT is uncertain. We examined the impact of omitting rectal preparation on prostate inter- and intrafraction motion, rectal volume, and their relationship.

**Methods:**

Two datasets were analysed. Dataset one included patients receiving RT for de novo prostate cancer on a C-arm linear accelerator, stratified by use of micro-enema or no rectal preparation. Interfraction prostate motion was calculated from daily CBCT, and compared between preparation groups, with further stratification by rectal volume on planning CT (pCT) < 90 or ≥ 90 cm^3^. Correlation between rectal volume and prostate motion was assessed.

Dataset two comprised 40 patients with localised prostate cancer treated without rectal preparation. Inter- and intrafraction prostate motion were calculated, with intrafraction motion assessed using post-treatment CBCT acquired at five RT fractions. The rectum was contoured on pre-treatment CBCT, and correlation between rectal volume and intrafraction motion evaluated.

**Results:**

Dataset one included 255 patients: 96 with and 159 without rectal preparation. Median rectal volume on pCT (proportion with rectal volume ≥ 90 cm^3^) was 64 cm^3^ (18 %) in the preparation group and 68 cm^3^ (28 %) in the no-preparation group. Interfraction motion differences were small; however, patients without preparation and a pCT rectal volume ≥ 90 cm^3^ demonstrated increased posterior motion.

In dataset two, median rectal volume on pCT was 75 cm^3^, 33 % had a rectal volume ≥ 90 cm^3^. Intrafraction motion was modest and not influenced by pCT rectal volume. No correlation was observed between rectal volume during treatment and intrafraction motion.

**Conclusion:**

Rectal preparation reduced the proportion of patients with a large rectum on pCT by only 10–15 %. A large rectum modestly increased interfraction motion but did not affect intrafraction motion. Findings suggest rectal preparation is not universally required with contemporary IGRT.

## Highlights

• Micro enema rectal preparation reduced the number of patients with a large rectum at planning CT by only 10–15 %
• Having a large rectum on planning CT, or during treatment, did not increase prostate intrafraction motion.
• Difference in prostate interfraction motion between preparation/no preparation groups was small.
• Results do not justify blanket use of rectal preparation for individuals undergoing radiotherapy to their prostate.

## Introduction

Prostate cancer (PCa) is the most frequently occurring male cancer in Europe [1]. In 2024 the number of men undergoing radical radiotherapy (RT) for PCa in England alone totalled almost 21,000 [2]. Demand for RT is anticipated to rise extensively, as European new cancer cases are expected to increase by 38 % by 2040 [3]. To meet demand, modern technology and techniques need to be used to full potential to improve RT efficiency and efficacy and achieve best patient outcomes.

Contemporary RT techniques for PCa involve the use of intensity modulation RT (IMRT) and volumetric modulated arc therapy (VMAT) coupled with daily online volumetric image guided RT (IGRT), aligning to the prostate gland to correct for interfraction motion [4], [5]. Where available online adaptive RT (oART) using CT- and MR-guided approaches can further enhance treatment precision with advanced imaging, real-time motion tracking, and on-table adaptive planning [6].

Despite having sophisticated RT strategies in place to treat PCa, many departments still impose strict rectal preparation regimes and rectal diameter constraints. These practices, essential to improve RT accuracy in the era of two-dimensional bone-based IGRT, where instigated by historical reports of large rectal volumes at planning causing excessive prostate motion, target underdosing, and higher biochemical failure rates [7], [8], [9]. IGRT innovations have since alleviated fears regarding PCa outcome [10], [11] yet burdensome preparation regimes remain.

Previously published findings supporting the relaxation of strict rectal diameter tolerances at the planning computer tomography (pCT) and advocated that rectal preparation was not necessary when delivering contemporary IGRT to the prostate [12]. Findings were however confounded by the fact that all patients analysed used micro enema rectal preparation prior to pCT and during RT. Since publishing we have modified our rectal preparation strategy, removing preparation for a subset of patients having radiotherapy to their prostate +/- seminal vesicles (see supplementary material A for rectal preparation stratification criteria). These changes prompted this follow up investigation which looks to comprehensively examine the impact of removing rectal preparation on prostate inter- and intrafraction motion, rectal volume, and correlation between the two.

## Method

Two patient datasets were investigated.

### Dataset one − stratified rectal preparation

Patients receiving PCa RT at The Royal Marsden NHS Foundation Trust between December 2023 and December 2024 were identified via review of pre-radiotherapy proformas, completed by patients as per standard clinical practice prior to RT. Data review and analysis was undertaken within service evaluation 1240, approved by the hospitals Committee for Clinical Research in February 2023.

Datasets eligible for motion analysis were defined as individuals receiving VMAT radiotherapy for de novo prostate cancer on a C-arm linac (Versa HD, Elekta). Four RT prescriptions were included; 36 Gy in 6-fractions once weekly, 36.25 Gy in 5-fractions alternate days, 57 Gy in 19-fractions daily and 60 Gy in 20-fractions daily. All patients followed the same bladder preparation regime; to empty their bladder one hour prior to pCT and treatment, and drink 350 ml of water to achieve a comfortable full bladder volume. The use of micro enema rectal preparation was dependant on RT prescription (Supplementary material A).

All patients were treated with cone beam CT (CBCT) image guidance, performed by two therapeutic radiographers, utilising dual registration to bone then implanted fiducial markers or prostate soft tissue, following an online no action level protocol. Offline, to determine interfraction independent prostate motion, isocentre deviations for bone and fiducial/soft tissue registrations were exported from Mosaiq (Elekta, Sweden) to Excel (Microsoft, USA). For each fraction, interfraction independent prostate motion in the lateral, longitudinal, and vertical direction was calculated by subtracting bone registration from fiducial/soft tissue registration values.

The dataset was divided into two groups, dependant on individuals use or not of enema preparation. Rescan rates instigated at the planning CT stage and during treatment were tallied. For each group, median, interquartile, and maximum interfraction motion range was calculated, tested for normality, and analysed using PRISM (Graphpad, USA). In each motion plane, patients with interfraction prostate motion greater than 5 mm for three or more fractions were classified as “movers”, and the proportion of “movers” calculated. The “movers” definition was informed by PACE trial guidance specifying a significant isocentre shift as > 5 mm [13], and the minimum requirement of three fractions to assess systematic error [4]. The proportion of fractions with motion greater than 5 mm was also calculated for each group.

The rectum was contoured on the pCT, from the rectosigmoid flexure to the anorectal junction, as per standard clinical practice on RayStation (RaySearch, Sweden). Enema and no enema groups were subdivided into; patients with a rectal volume on pCT < 90 cm^3^ and patients with a rectal volume on pCT ≥ 90 cm^3^. The large rectum classification, volume ≥ 90 cm, was established from the literature [9]. Subgroup interfraction motion and “mover” analysis was undertaken as per the prior presented methodology.

### Dataset two – No rectal preparation

The second dataset was prospectively sampled, as approved by The Royal Marsden clinical audit board (RT98-2024), to enable intrafraction motion analysis. This cohort included 40 patients with localised PCa, receiving RT May-September 2024. Eligible individuals had:

• No rectal preparation
• Bladder preparation as previously described
• RT to prostate and proximal seminal vesicles, 60 Gy in 20-fractions
• VMAT on a c-arm linac (Versa HD, Elekta, Sweden)
• Daily CBCT-guidance, dual registration to bone then implanted fiducials or prostate soft-tissue, following an online no action level protocol
• Post-treatment CBCT fractions 1, 5, 10, 15 and 20

Approved radiotherapy plans were examined, to consider plan quality the number of target and organs at risk mandatory and optimal dose constraints met were recorded. Rescan rates instigated at the pCT stage and during treatment were also tallied.

Interfraction independent prostate motion was calculated as per prior presented methodology. Intrafraction motion was the deviation in prostate position on post-treatment CBCT from planned, after pre-treatment motion correction. Post treatment CBCTs were registered online by two therapeutic radiographers. Independent prostate motion was calculated by subtracting bone registration values from fiducial/soft-tissue displacements. Median, interquartile, and maximum inter- and intrafraction motion range was calculated, tested for normality and analysed using PRISM (Graphpad, USA). Intrafraction time was measured from the start of the pre-treatment CBCT to the start of the post-treatment CBCT, timing data was taken directly from Mosaiq (Elekta, Sweden).

Rectum and bladder were delineated on patient’s radiotherapy pCT and approved as per standard clinical practice in RayStation (RaySearch, Sweden). In addition, rectum and bladder were contoured on pre-treatment CBCT for fractions 1, 5, 10, 15, and 20 in RayStation by one of four competent therapeutic radiographers (SA, LB, AG, HM) and checked by a second. Group median rectal and bladder volume and change in median volume during RT were calculated for the whole group and for the group subdivided by rectal volume on pCT < 90 cm^3^ and ≥ 90 cm^3^. Rectum content was reviewed, rectal gas was delineated using volume thresholding, and this volume divided by the total rectal volume to give percent gas volume.

## Results

### Dataset one − stratified rectal preparation

Interfraction motion data was calculated for 255 patients: 96 using and 159 not using enema preparation. Twenty-one (8 %) of these patients required a rescan at the pCT stage, instigated as their rectal diameter was larger than the predefined maximum threshold of 4.5 cm with or 5.0 cm without enema preparation, on the initial scan. A slightly larger proportion of patients required a rescan in the no enema group (Table 1). During radiotherapy three patients required a rescan due to changes in rectal volume preventing accurate radiotherapy delivery, two in the enema and one in the no enema group.

**Table 1:** Motion and mover results for dataset one.

	**Enema**						**No Enema**					
Number of patients (%)	131 (44)						165 (56)					
Number (%) patients requiring a rescan at planning CT	7 (5)						14 (8)					
Median (range) rectal volume	64 (27–185)						68 (33–194)					
Patients (fractions) with motion data	96(1747)						159 (2583)					
Median (IQR) motion (mm)	**LAT**		**LONG**		**VERT**		**LAT**		**LONG**		**VERT**	
	**0.1** (−0.5–0.7)		**0.2** (−0.9–1.6)		**0.3** (−1.5–2.3)		**0.1** (−0.4–0.5)		**0.2** (−1.1–1.8)		**0.1** (−1.7–2.0)	
Number (%) of “movers” (motion > 5 mm for ≥ 3 fractions)	**1 (1)**		**13 (14)**		**25 (26)**		**2 (1)**		**26 (16)**		**49 (31)**	
Number (%) fractions with motion > 5 mm	**17 (1)**		**88 (5)**		**197 (11)**		**15 (1)**		**241 (10)**		**371 (14)**	
**Sub-group analysis**	**Rectum < 90 cm^3^ at CT**			**Rectum ≥ 90 cm^3^ at CT**			**Rectum < 90 cm^3^ at CT**			**Rectum ≥ 90 cm^3^ at CT**		
Number of subgroup patients (%)	107 (82)			24 (18)			119 (72)			46 (28)		
Median (range) rectal volume cm^3^	57 (27–89)			111 (91–185)			60 (33–89)			111 (90–194)		
Patients (fractions) with motion data	82 (1524)			14 (224)			115 (1946)			44 (636)		
Median (IQR) motion (mm)	LAT	LONG	VERT	LAT	LONG	VERT	LAT	LONG	VERT	LAT	LONG	VERT
	**0.1** (−0.4–0.7)	**0.3** (−0.9–1.6)	**0.4** (−1.3–2.4)	**−0.2** (−0.9–0.4)	**0.0** (−1.0–1.3)	**−0.3** (−2.2–1.1)	**0.1** (−0.3–0.5)	**0.2** (−1.5–1.8)	**0.3** (−1.4–2.3)	**0.0** (−1.4–0.5)	**0.4** (−0.5–2.08)	**−0.6** (−3.0–1.2)
Number (%) of “movers” (motion > 5 mm for ≥ 3 fractions)	**1** **(1)**	**11 (13)**	**23 (28)**	**0** **(0)**	**3** **(21)**	**2** **(14)**	**1** **(1)**	**19** **(17)**	**34** **(30)**	**1** **(2)**	**7** **(16)**	**14** **(32)**
Number (%) fractions with motion > 5 mm	**12** **(1)**	**75** **(5)**	**177** **(12)**	**5** **(2)**	**16** **(7)**	**20** **(9)**	**8** **(1)**	**170** **(9)**	**243** **(12)**	**7** **(1)**	**69** **(11)**	**116** **(18)**

Table 1 presents motion results divided by enema use and further subdivided by rectal volume on planning CT < 90 cm^3^ or ≥ 90 cm^3^. Seventy patients (27 %) had a rectal volume ≥ 90 cm^3^ on planning CT. The proportion of patients meeting the large rectum threshold was greater in the no-enema group.

**Fig. 1:**
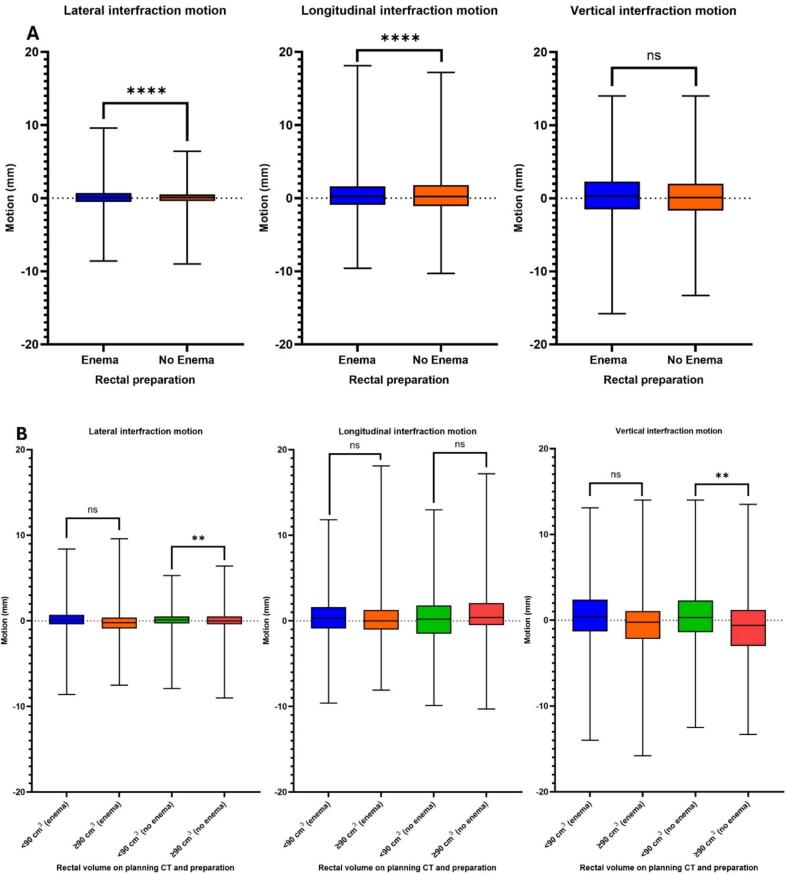
(A) Interfraction independent prostate motion for individuals grouped by enema preparation yes/no. (B). Interfraction independent prostate motion for individuals grouped by enema preparation yes/no and sub-grouped by rectal volume < 90 cm^3^ or ≥ 90 cm^3^. p-values calculated on absolute difference, ns = not significant, ** = p < 0.01, *** = p < 0.001, **** = p < 0.0001.

Differences in interfraction motion between the enema and no enema group were small. Motion data was not normally distributed, a Mann-Whitney test (significant *p* < 0.05), calculated on absolute values, presented a significant difference between groups in the lateral and longitudinal direction (Fig. 1A). These significant differences, based on a ranked *p* value, are however clinically insignificant. Fig. 1B presents motion for patients grouped by enema use and sub-grouped by rectal volume on pCT < 90 cm^3^ or ≥ 90 cm^3^. The most notable result is a significant difference in vertical motion, seen in the no enema group. Individuals with large rectums at pCT had more negative (posterior) interfraction motion, likely due to reduced rectal volume during RT. Even though significant, the difference in median vertical motion between no enema patients with rectal volumes on pCT < 90 cm^3^ or ≥ 90 cm^3^ remained under 1 mm.

The proportion of “movers” was similar for both groups although marginally higher in the vertical direction for the no enema group (31 vs 26 %). When sub-divided by rectal volume at pCT < or ≥ 90 cm^3^ no impact on mover status is seen in the enema group, and only small differences in the no enema group. The percentage of fractions with prostate motion > 5 mm was also small, although slightly greater in the no enema group (see supplementary material B for graphical representation of this data).

### Dataset two − no rectal preparation

No patients in this group required a rescan at the pCT stage or during radiotherapy. A clinically acceptable plan was achieved for all patients and approved for treatment by the clinical team as per standard practice. Each plan was assessed against 36 clinical goals (see supplementary material C). The radiotherapy plans of 30 patients achieved all optimal dose constraints. Eight patient plans met 35 optimal and one mandatory dose constraint; one patient plan met 34 optimal and 2 mandatory dose constraints and one patient plan met 33 optimal and three mandatory dose constraints. Optimal constraints not met were PTVpsv_4700 D99 % ≥ 44.650 Gy (3 patients), bladder V56.8 Gy ≤ 35 % (2 patients) and penile bulb V22Gy ≤ 50 % (1 patient), all remained within mandatory dose limits. From the 10 patients not meeting all optimal dose criteria, four had a rectal volume ≥ 90 cm^3^ on planning CT.

**Fig. 2:**
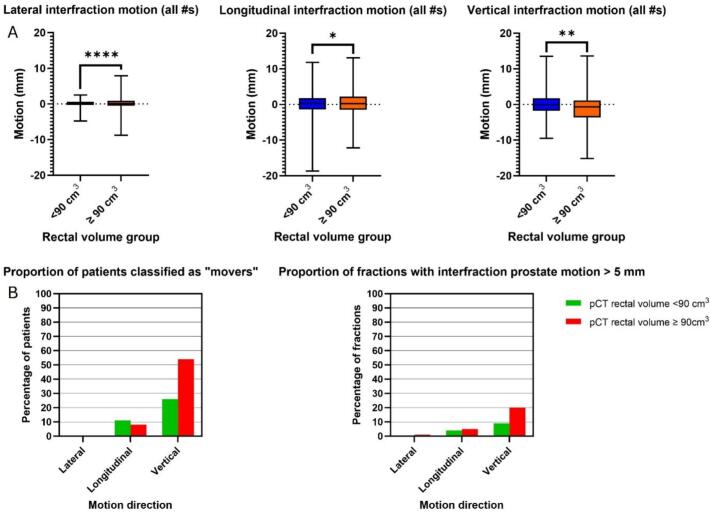
(A). Interfraction independent prostate motion for individuals grouped by rectal volume on pCT < 90 cm^3^ or ≥ 90 cm^3^. p-values calculated on absolute difference, * = p < 0.05, ** = p < 0.01, **** = p < 0.0001. (B) On the left, the proportion of patients meeting the classification of “mover” in each direction, and on the right the proportion of fractions with interfraction motion > 5 mm.

Table 2 presents inter- and intrafraction motion results and “mover” status for the whole group (n = 40) and separated by rectal volume on pCT < 90 cm^3^ or ≥ 90 cm^3^. Motion data was not normally distributed, a Mann-Whitney test (significant p < 0.05), calculated on absolute values, presented significant differences in all directions (Fig. 2A). Lateral and longitudinal motion differences are clinically insignificant, but as per dataset one, individuals with large rectums at pCT had more negative (posterior) interfraction motion.

**Table 2:** Motion and mover results for dataset two.

**Interfraction motion (n = 800 #s)**						
**Median (IQR) interfraction motion (mm)**	**Lateral**		**Longitudinal**		**Vertical**	
	0.1 (−0.4, 0.6)		0.3 (−1.4, 1.9)		−0.3 (−2.2, 1.4)	
**Number (%) of “movers” (motion > 5 mm for ≥ 3 fractions)**	0 (0)		4 (10)		14 (35)	
**Number (%) fractions with motion > 5 mm**	2 (0)		35 (4)		99 (12)	
**Sub-group**	**Rectum < 90 cm^3^ at pCT**			**Rectum ≥ 90 cm^3^ at pCT**		
**Number of patients (%)**	27 (67)			13 (33)		
**Median (IQR) interfraction motion (mm)**	**LAT**	**LONG**	**VERT**	**LAT**	**LONG**	**VERT**
	0.1 (−0.3, 0.6)	0.3 (−1.4, 1.8)	−0.1 (−1.8, 1.7)	0.1 (−0.5, 0.9)	0.3 (−1.5, 2.2)	−0.7 (−3.6, 1.1)
**Number (%) of “movers” (motion > 5 mm for ≥ 3 fractions)**	0 (0)	3 (11)	7 (26)	0 (0)	1 (8)	7 (54)
**Number (%) fractions with motion > 5 mm**	0 (0)	23 (4)	47 (9)	2 (1)	12 (5)	52 (20)
**Intrafraction motion (n = 198 #s)**						
**Median (IQR) interfraction motion (mm)**	**Lateral**		**Longitudinal**		**Vertical**	
	0.2 (−0.3, 0.7)		0.3 (−1.6, 1.8)		−0.3 (−2.3, 1.5)	
**Number (%) fractions with motion > 3 mm**	2 (1)		58 (29)		63 (32)	
**Sub-group**	**Rectum < 90 cm^3^ at pCT (n = 134 #s)**			**Rectum ≥ 90 cm^3^ at pCT (n = 64 #s)**		
**Median (IQR) interfraction motion (mm)**	**LAT**	**LONG**	**VERT**	**LAT**	**LONG**	**VERT**
	0.1 (−0.2, 0.7)	0.3 (−1.6, 2.0)	−0.3 (−1.7, 2.1)	0.2 (−0.4, 0.7)	0.3 (−1.7, 1.7)	−0.4 (−3.6, 1.1)
**Number (%) fractions with motion > 3 mm**	2 (1)	46 (34)	42 (31)	0 (0)	12 (19)	31 (33)

**Fig. 3:**
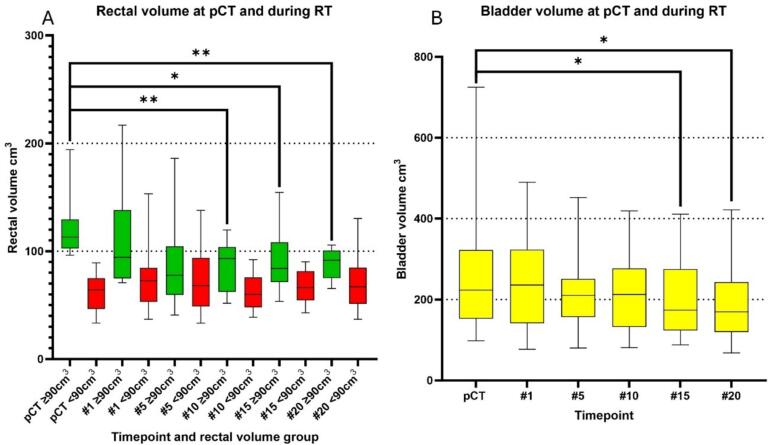
(A) Rectal volume at pCT and throughout RT grouped by rectal volume on pCT < 90 cm^3^ (red) or ≥ 90 cm^3^ (green). p-values * = p < 0.05, ** = p < 0.01. (B) Bladder volume at pCT and throughout RT for all dataset two patients. p-values * = p < 0.05. (For interpretation of the references to colour in this figure legend, the reader is referred to the web version of this article.)

For this dataset, rectal volume changes during RT were quantified, results support our prior interpretation that the posterior shift is attributed to reducing rectal volume during RT, seen only in the group with a rectal volume on pCT ≥ 90 cm^3^ (Fig. 3A). For those with pCT rectal volume ≥ 90 cm^3^, median (range) rectal volume was 113 (96–194) cm^3^, with gas accounting for 7 % (0–25 %) of content (supplementary material D). Median (range) rectal volume was 64 (33–89) cm^3^ in the < 90 cm^3^ group, combining both groups gives a median (range) rectal volume at pCT of 75 (33–194) cm^3^. Significant reduction in bladder volume was also seen in the last 1–2 weeks of RT, median (range) volume at pCT was 224 (98–727) cm^3^ dropping to 169 (68–422) cm^3^ on the last fraction of RT (Fig. 3B).

The proportion of patients classified as “movers” was akin to dataset one (35 vs 31 %) but the proportion of “movers” with a rectal volume ≥ 90 cm^3^ at pCT was larger (54 vs 32 %). This result may have been skewed by the small size of this group, n = 13. The percentage of fractions with prostate motion > 5 mm was less than dataset one in the superior/inferior direction but slightly greater in the vertical direction for the no enema group (Fig. 2B).

**Fig. 4:**
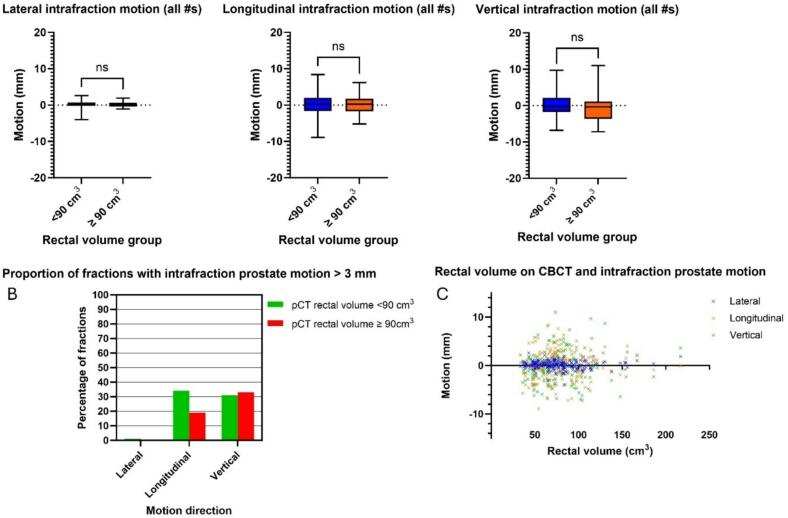
(A) Intrafraction independent prostate motion for individuals grouped by rectal volume on pCT < 90 cm^3^ or ≥ 90 cm^3^. *p-values calculated on absolute difference, ns = not significant*. (B) The proportion of fractions with intrafraction motion > 3 mm. (C) Rectal volume on CBCT and intrafraction prostate motion for that fraction, in all three directions.

The key finding was what intrafraction motion was modest (Fig. 4A) and not impacted by rectal volume on pCT being < 90 cm^3^ or ≥ 90 cm^3^. The proportion of fractions displaying motion greater than 3 mm was similar between both rectal volume groups (Fig. 4B). No significant correlation (Spearman’s rho) between patients on treatment rectal volume and intrafraction motion for that fraction was found (Fig. 4C); lateral rs (193) = -0.09, p 0.23; longitudinal rs (193) = 0.12, p 0.10; vertical rs (193) = 0.11, p 0.12.

Mean (1SD) intrafraction time, from starting the pre-treatment CBCT to starting the post-treatment CBCT was 6 min 36 s (1 min 10 s).

## Discussion

In the absence of enema preparation 28–33 % of patients had a large rectum (>90 cm^3^) on their pCT. With enema preparation the proportion was 18 % (dataset 1) suggesting that enema use helped avoid a large rectum in 10–15 % of individuals. In support of these findings, we previously reported that 13 % of PCa patients, all using enema preparation, triggered a repeat pCT due to having a distended rectal diameter on initial pCT (> 4.5 cm anterior posterior) [14], compared to a rate of 31 %, in a prior patient group not using enemas, audited under the same criteria (unpublished internal audit, 2011). We must emphasise however that comparisons here are made across heterogeneous patient groups, with varying patient demographics, tumour staging, RT prescriptions and means of defining a large rectum, variations in rectal volume cannot therefore be attributed solely to enema use.

Still, this finding raised the question, should we reintroduce a blanket enema prescription for all patients? We believe the data does not support this, rather that patient management should be personalised. This is supported by the 10.13039/100011010ESTRO ACROP consensus guideline which does not recommend preparation as routine practice [5]. Justification for our decision is discussed herein.

Rescan rates at the pCT stage and during treatment remained low without enema preparation, and clinically acceptable plans were achieved.

Prostate interfraction motion, without enema preparation, remained small and akin to the motion measured in those using enemas. Interfraction motion, including greater posterior motion for those with larger rectal volumes on pCT, can be corrected for with contemporary online IGRT protocols [4], [5]. Our limited review of plan dosimetry on pCT does not consider the impact of daily anatomical changes on delivered dose. However previous work showed that when disease is localised to the prostate gland, the application of translational corrections can maintain acceptable dose to the prostate, even when rectal volume changes occur [15]. However, changes in rectal volume from pCT, can result in significant underdosing of the seminal vesicles [15], [16], [17], which rotate independent of the prostate gland [18], [19], [20], [21]. Consequently, for individuals where prostate cancer has invaded the seminal vesicles (T3b disease) or there is a high risk of seminal vesicle involvement, having a large rectal volume on pCT may have greater detriment on patient outcomes [22], [23]. Considering this, we recommend intervention for patients with both locally advanced prostate cancer and a rectal volume ≥ 90 cm^3^ on pCT. One intervention could be to repeat the pCT with the addition of rectal preparation. This option is straightforward but relies on CT availability, which if limited may delay the patient pathway. There is also no clear evidence on the effectiveness of one rectal preparation regime over another [24], so the addition of preparation is not guaranteed to reduce rectal volume. As this work revealed, large rectal volumes still present, even with enema preparation, and rescanning with additional preparation may offer limited benefit [14].

An alternative solution, and our preferred solution, is to redirect individuals meeting this criterion to an online adaptive RT (oART) pathway. The application of MRI-guided oART was shown to optimise delivered RT dose to the prostate and seminal vesicles even when large changes in rectal volume occurred [15], [25], [26]. CT-guided systems have also been shown to achieve this [27], [28]. This approach negates the need for rescanning or additional preparation but does rely on hospitals having an oART pathway in place.

Current capacity to deliver oART is limited internationally but provisions are rapidly rising. Efficient and effective utilisation of this technology is needed to warrant its additional cost and resource requirements [6], [29], [30]. Implementing stratification criteria, as suggested in this paper, aims to improve efficacy and achieve greatest patient benefit from oART for individuals with PCa. This approach is most pertinent for tumour sites with high patient numbers, like PCa, where limited oART capacity means it is infeasible to offer it to all patients. Twenty percent of patients in dataset two met our stratification criteria, delivering oART to this volume of patients with PCa is more achievable.

With respect to intrafraction motion, our findings support previous work which reported that a large rectal volume on pCT or during RT treatment did not influence intrafraction motion [12]. Prior work sampled patients using micro enema preparation, it is therefore reassuring to now report the same findings in patients not using rectal preparation. Unfortunately, differences in methodology preclude direct comparison. True intrafraction motion may indeed be lower than our reported findings as the measurement methodology followed, using pre- and post-treatment CBCT, can include motion after treatment cessation [31]. However, in the absence of online tracking solutions, it remains a valid methodology.

Prostate intrafraction motion variance has been reported to increase over time, following a random walk model [32], [33], [34]. Treatment times for individuals in dataset two were swift, averaging 6 min 36 s, our results should therefore not be extrapolated to substantially longer treatments. Rectal content has also been shown to impact intrafraction motion, with only gas having a significant impact [35]. In dataset two, large rectal volumes at pCT were mostly stool filled with small volume gas; therefore, caution is still warranted for exceptionally gaseous cases. A limitation of our study is that rectal content was not analysed on CBCT imaging. Another limitation of this study is that we did not capture patient toxicity or wellbeing data alongside rectum and bladder volume. As a result, we cannot definitively say why rectal volume reduced from fraction 10 onwards in those with a pCT rectal volume ≥ 90 cm^3^, but not in those who did not reach this threshold at pCT. It is plausible that rectal volume reductions, due to acute proctitis or variations in patient’s routine e.g. increased hydration or activity, simply appear more obvious in those with a large rectum initially. We can also not comment on the impact of rectal preparation on toxicity.

Omitting routine use of enemas also has cost and environmental benefits. The cost of a 12 pack of micro enemas in the UK, in 2025 was £8.99 [36]. As our RT department delivers approximately 600 courses of PCa RT annually, our previous blanked prescription of 15 enemas per patient would amass to £6,743. With healthcare budgets of increasing concern at every governmental level across Europe [37], cost savings such as this, are encouraged. Organisations must also be mindful of waste management and the associated environmental impact [38]. Each enema is administered via a plastic tube and nozzle, which patients discard in domestic or clinical waste. Reduced enema use is therefore directly correlated with less waste production.

## Conclusion

Micro enema rectal preparation for PCa RT reduced the number of patients with a large rectum at pCT by 10–15 %. We do not believe this reduction warrants the blanket use of bowel preparation for all patients, as having a large rectum (≥90 cm^3^) on pCT, or during RT, did not increase prostate intrafraction motion. Having a large rectum on pCT did increase the risk of prostate interfraction motion in the vertical plane, but this change was modest and can largely be accounted for with contemporary online image-guidance.

Interfraction changes in rectal volume can lead to significant underdosing of seminal vesicles therefore we do recommend further intervention for patients with both a large rectum on pCT and seminal vesicle involvement, or a high risk of seminal vesicle involvement. Stratifying patients meeting this criterion to oART would optimise RT dose delivered for the individual, whilst also using oART to best effect to achieve greatest patient benefit for individuals with PCa.

## Declaration of Competing Interest

The authors declare that they have no known competing financial interests or personal relationships that could have appeared to influence the work reported in this paper.

## Appendix A Supplementary data

Supplementary Data 1 Supplementary data A-D includes enema stratifcation criteria, dataset 1 "movers" and interfraction motion > 5 mm graphs, dose contrainsts for 60Gy in 20-fraction PCa RT and dataset 2 rectal gas analysis.
