# Supplementary material for: Is rectal preparation necessary in contemporary image-guided prostate radiotherapy?
Source: Clin Transl Radiat Oncol. 2026 Jun 2;60:101207. doi: 10.1016/j.ctro.2026.101207 (PMC13276509; doi:10.1016/j.ctro.2026.101207)
Supplement: Supplementary Data 1 — Supplementary data A-D includes enema stratifcation criteria, dataset 1 "movers" and interfraction motion > 5 mm graphs, dose contrainsts for 60Gy in 20-fraction PCa RT and dataset 2 rectal gas analysis. [file mmc1.docx]

**Supplementary material**

1. Patient groups using / not using enemas for prostate cancer radiotherapy preparation


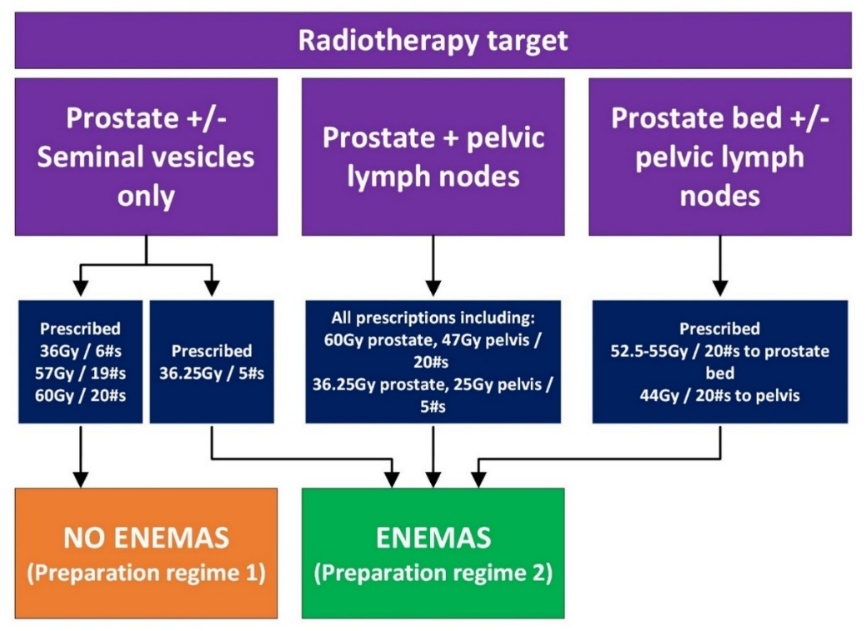


1. On the left, the proportion of patients meeting the classification of “mover” in each direction, and on the right the proportion of fractions with interfraction motion > 5 mm, for those using and not using enema preparation.


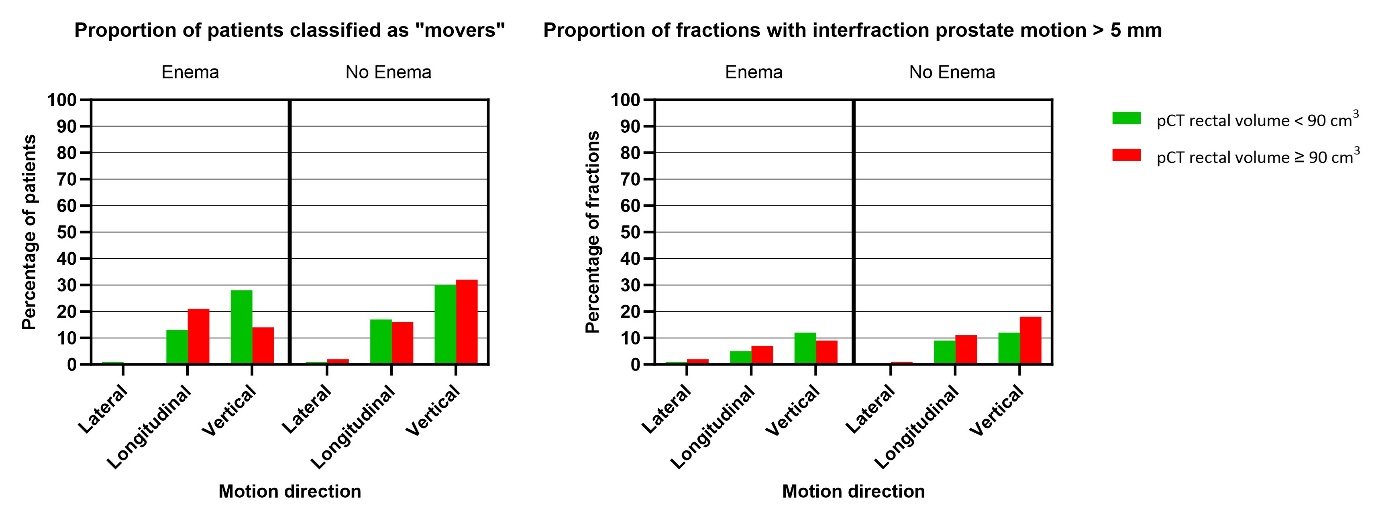


1. The 36 optimal clinical goals implemented for 60Gy in 20# prostate radiotherapy at our Institution

| **Structure** | **Constraint** |
| --- | --- |
| PTVp_6000 | D50.00 % + 60.600 Gy |
| PTVp_6000 | D50.00 % ≥ 59.400 Gy |
| PTVp_6000 | D99.00 % ≥ 57.000 Gy |
| PTVp_6000 | D1.00 % ≤ 63.000 Gy |
| PTVpsv_4700 | D99.00 % ≥ 44.650 Gy |
| Rectum | V40.000 Gy ≤ 60.00 % |
| Rectum | V48.000 Gy ≤ 50.00 % |
| Rectum | V50.000 Gy ≤ 30.00 % |
| Rectum | V56.000 Gy ≤ 15.00 % |
| Rectum | V60.000 Gy ≤ 3.00 % |
| Rectum | V20.000 Gy ≤ 85.00 % |
| Rectum | V30.000 Gy ≤ 57.00 % |
| Rectum | V40.000 Gy ≤ 38.00 % |
| Rectum | V50.000 Gy ≤ 22.00 % |
| Rectum | V60.000 Gy ≤ 1.00 % |
| Bladder | V48.000 Gy ≤ 50.00 % |
| Bladder | V56.800 Gy ≤ 35.00 % |
| Bladder | V40.000 Gy ≤ 50.00 % |
| Bladder | V48.000 Gy ≤ 25.00 % |
| Bladder | V56.800 Gy ≤ 5.00 % |
| Bladder | V60.000 Gy ≤ 25.00 % |
| Bladder | V60.000 Gy ≤ 3.00 % |
| FemoralHead_L | V40.500 Gy ≤ 5.00 % |
| FemoralHead_L | V40.500 Gy ≤ 50.00 % |
| FemoralHead_R | V40.500 Gy ≤ 5.00 % |
| FemoralHead_R | V40.500 Gy ≤ 50.00 % |
| Bowel | V36.000 Gy ≤ 158.00 cm³ |
| Bowel | V40.000 Gy ≤ 110.00 cm³ |
| Bowel | V44.000 Gy ≤ 28.00 cm³ |
| Bowel | V48.000 Gy ≤ 6.00 cm³ |
| Bowel | V52.000 Gy ≤ 0.01 cm³ |
| Bowel | V36.000 Gy ≤ 78.00 cm³ |
| Bowel | V40.000 Gy ≤ 40.00 cm³ |
| Bowel | V44.000 Gy ≤ 14.00 cm³ |
| Bowel | V48.000 Gy ≤ 0.50 cm³ |
| PenileBulb | V22.000 Gy ≤ 50.00 % |

1. Dataset two, large rectum (>90cm^3^) subgroup: volume of rectal gas analysis on pCT

| **Patient number** | **Rectum length on pCT (cm)** | **Rectum volume on pCT (cm^3^)** | **Rectal gas volume on pCT (cm^3^)** | **% of pCT rectal volume which is gas** |
| --- | --- | --- | --- | --- |
| **3** | **7.8** | **112** | **21** | **19%** |
| **5** | **11.0** | **128** | **10** | **8%** |
| **10** | **11.0** | **103** | **5** | **5%** |
| **14** | **9.8** | **123** | **8** | **7%** |
| **17** | **9.8** | **104** | **7** | **7%** |
| **18** | **9.2** | **101** | **5** | **5%** |
| **19** | **11.1** | **130** | **0** | **0%** |
| **22** | **12.3** | **113** | **12** | **11%** |
| **30** | **8.4** | **96** | **2** | **2%** |
| **31** | **10.7** | **131** | **8** | **6%** |
| **33** | **12.0** | **194** | **2** | **1%** |
| **34** | **12.4** | **122** | **31** | **25%** |
| **39** | **8.2** | **102** | **9** | **9%** |
